# Protein persulfidation: a ubiquitous modification regulating a broad spectrum of biological processes

**DOI:** 10.1093/jxb/eraf464

**Published:** 2025-10-18

**Authors:** Luis C Romero, Reyes Carrillo, David Montesinos-Pereira, Carmen Luque, Angeles Aroca, Cecilia Gotor

**Affiliations:** Instituto de Bioquímica Vegetal y Fotosíntesis, Consejo Superior de Investigaciones Científicas and Universidad de Sevilla, Avenida Américo Vespucio, 49, Seville 41092, Spain; Instituto de Bioquímica Vegetal y Fotosíntesis, Consejo Superior de Investigaciones Científicas and Universidad de Sevilla, Avenida Américo Vespucio, 49, Seville 41092, Spain; Instituto de Bioquímica Vegetal y Fotosíntesis, Consejo Superior de Investigaciones Científicas and Universidad de Sevilla, Avenida Américo Vespucio, 49, Seville 41092, Spain; Instituto de Bioquímica Vegetal y Fotosíntesis, Consejo Superior de Investigaciones Científicas and Universidad de Sevilla, Avenida Américo Vespucio, 49, Seville 41092, Spain; Instituto de Bioquímica Vegetal y Fotosíntesis, Consejo Superior de Investigaciones Científicas and Universidad de Sevilla, Avenida Américo Vespucio, 49, Seville 41092, Spain; Instituto de Bioquímica Vegetal y Fotosíntesis, Consejo Superior de Investigaciones Científicas and Universidad de Sevilla, Avenida Américo Vespucio, 49, Seville 41092, Spain; Estación Experimental del Zaidín, Spain

**Keywords:** Development, hydrogen sulfide, metalloproteins, methylation, proteolysis, proteomics, stress responses, thioredoxin system

## Abstract

Hydrogen sulfide signaling occurs mainly through protein persulfidation, an important post-translational modification and a highly dynamic process in plants. Beyond enzyme activity, persulfidation affects protein localization, structure, and interactions. This review provides an overview of the mechanisms governing this modification and the pivotal role of the thioredoxin system mediating depersulfidation, which is essential for maintaining redox homeostasis. Proteomic studies in *Arabidopsis thaliana* and crop species reveal that protein persulfidation is a widespread post-translational modification in plants, with over 11 700 polypeptides identified as susceptible. Functional analysis shows a high proportion of these proteins are involved in primary metabolic pathways and secondary metabolism. Gene Ontology analysis highlights additional processes regulated by persulfidation, underscoring its broad regulatory role in plant biology. These processes are mainly related to proteolysis, defense response, embryo development, protein transport, and response to cold. In addition, other processes regulated by hydrogen sulfide, which have been widely studied, also stand out, such as response to abscisic acid, response to oxidative stress, and response to water deprivation. A comprehensive description of the persulfidated proteins implicated in the processes highlighted by the Gene Ontology analysis is provided. This integrated role of H_2_S with other molecules offers a panoramic overview of its importance in plant biology which also helps to raise new questions and working directions for the future research.

## Teaser

This review underscores the critical role of protein persulfidation in regulating different aspects of plant biology, emphasizing metalloproteins as important targets and thioredoxin system for depersulfidation.

## Introduction

Hydrogen sulfide (H_2_S) is a molecule that exhibits dual behavior like other signaling molecules such as nitric oxide, hydrogen peroxide, and carbon monoxide. As with these other molecules, above a specific concentration threshold H_2_S becomes toxic, in this case because it inhibits the mitochondrial respiratory chain at the level of cytochrome c oxidase. On the contrary, below this concentration threshold, H_2_S acts as a signaling molecule, becoming an essential regulator of numerous physiological processes in both mammals and plants (Filipovic *et al*., 2018; Gotor *et al*., 2019; Aroca *et al*., 2020; Zhang *et al*., 2021; Aroca and Gotor, 2022b).

In recent years, there has been an exponential increase in research on H_2_S signaling in plants, making it a prominent area of study. H_2_S regulates essential processes for plant performance, being involved in developmental programs such as seed germination and root development. It also plays a fundamental role in leaf senescence, stomatal movement and development, and autophagy (Aroca *et al*., 2018; Pantaleno *et al*., 2021; Aroca and Gotor, 2022a; Huang and Xie, 2023; Jaiswal *et al*., 2025). H_2_S also regulates plant responses to adverse environmental conditions, thereby enhancing their resilience. Recently, in the current context of climate change, H_2_S-mediated regulation of these responses has become the focus of intensive research (Corpas, 2019; Aroca *et al*., 2021b; Aroca *et al*., 2023; Mingjian *et al*., 2025).

H_2_S signaling occurs primarily through a post-translational modification (PTM) called persulfidation, which is the result of the modification of cysteine (Cys) residues in proteins to form persulfide groups. In this review, we summarized the current research on protein persulfidation in plants. The molecular mechanisms of this modification are described, along with the possible functional consequences and effects on growth, plant metabolism, and adaptation to abiotic stress. Together, these data highlight the multilayered importance of H_2_S-dependent persulfidation in plant physiology and adaptation.

## The multifaceted routes to cysteine persulfidation

The post-translational modification of cysteine residues to give persulfides (Cys-SSH) has been recognized as a regulatory mechanism among various kingdoms including plants. This reversible and dynamic modification has the potential to change protein conformation and to regulate variety of cellular processes. Thus, persulfidation is of significance in regulation of protein function, enzyme activity, and protein–protein interactions, impacting physiological process and responses to stress. Protein persulfides are formed by a variety of independent and related pathways which collectively regulate the intricate signaling network of H_2_S.

The main enzymes that participate in H_2_S biosynthesis in plants, including L-cysteine desulfhydrase (LCD), D-cysteine desulfhydrase (DCD), sulfite reductase (SiR), and β-cyanoalanine synthase (CAS), are located in different cellular compartments, such as cytosol, chloroplasts, and mitochondria (Aroca *et al*., 2018; Aroca *et al*., 2020). The coordinated function of these enzymes and their subcellular localization ensures a fine-tuned regulation of H₂S concentration by an environmental perturbation or particular signal. The expression or the enzymatic activity of endogenous H₂S-generating enzymes may be induced under specific conditions and provoke the subsequent increase of intracellular H_2_S concentration.

**Fig. 1.**
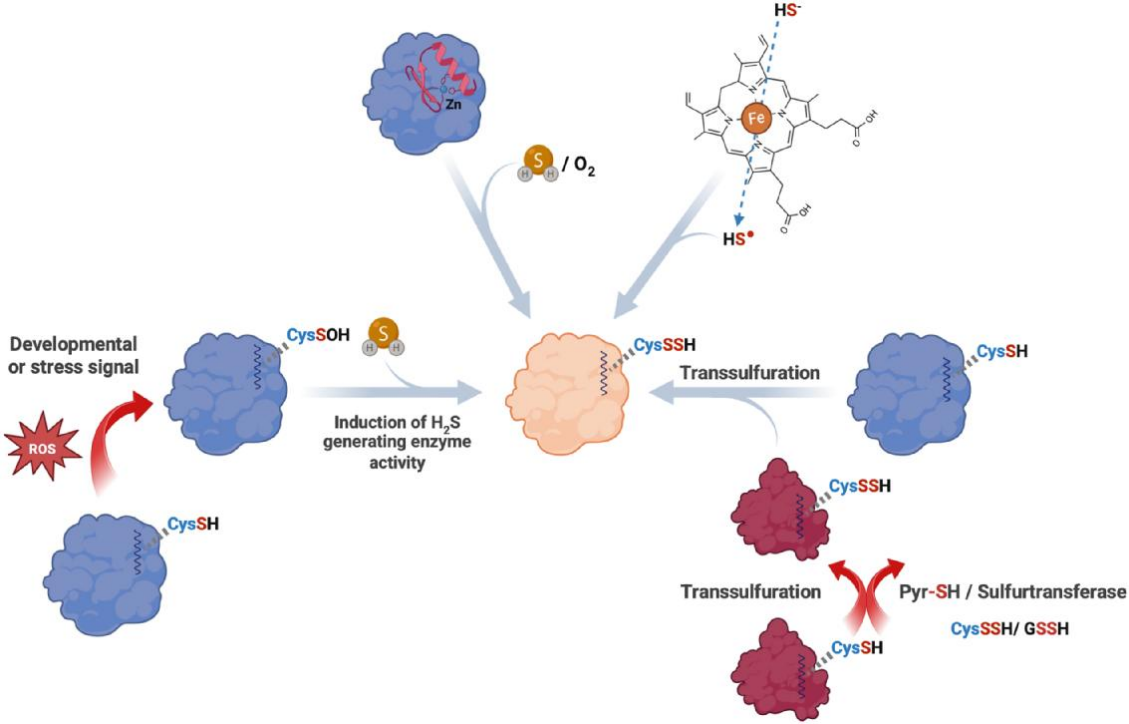
Mechanism of action for hydrogen sulfide(H₂S) in protein persulfidation. The interaction of H₂S with oxidized cysteine species, including sulfenic acids (Cys-SOH), is the best established process. A second mechanism for protein persulfide formation is through transsulfuration reactions, consisting of the donation of a sulfur atom from a sulfur compound catalyzed by sulfurtransferases, or the exchange with low-molecular-weight persulfides like persulfide of cysteine (CysSSH) and glutathione (GSSH). A non-enzymatic mechanism may involve metal elements within metalloproteins, such as Zinc-finger motifs or metal cofactors including heme iron, copper, or iron-sulfur clusters. Created in BioRender. Aroca, A. (2025) https://BioRender.com/fm2nhwp.

One established process for the formation of persulfides is the interaction of H₂S, an endogenous gasotransmitter, with oxidized cysteine species including sulfenic acids (Cys-SOH) (Fig. 1) (Aroca *et al*., 2015; Aroca *et al*., 2021b). This pathway indicates an important crosstalk between reactive oxygen species (ROS) and reactive sulfur species (RSS) signaling, in which H₂O₂-mediated sulfenylation can act as a prerequisite for the following persulfidation, as has been reported in various biological systems (Zivanovic *et al*., 2019; Garcia-Calderon *et al*., 2023).

This crosstalk involves two post-translational modifications (PTMs) of proteins on cysteine residues, the oxidative PTM, sulfenylation (R−SOH), and the sulfur-based PTM, persulfidation (R−SSH). Sulfenylation, the reversible oxidation of a cysteine thiol (R−SH) by hydrogen peroxide (H_2_O_2_), acts as a prerequisite for the formation of persulfides. This is because H_2_S cannot directly react with the reduced thiol group (R−SH) of a protein. Instead, it reacts with the oxidized sulfenic acid intermediate (R−SOH) to form the persulfide (R−SSH) and a water molecule. Therefore, the formation of the sulfenic acid during the first stage of oxidation increases the electrophilic character of the sulfur, allowing it to be readily targeted for nucleophilic attack by H₂S or other sulfane sulfur species leading to persulfide formation (Aroca *et al*., 2021b). Thus, under stress conditions, an increase in sulfenylation often precedes a subsequent, shifted increase in persulfidation, creating a ‘shifted wave’ of redox modifications that fine-tunes protein activity and cellular responses to environmental cues.

Transsulfuration is also a major route for persulfidation of proteins (Fig. 1). This mechanism involves donation of a sulfur atom from one persulfide to another thiol group-containing substrate. Sulfurtransferases, like 3-mercaptopyruvate sulfurtransferase (3-MST), are enzymes that have been shown to perform this reaction. For example, a protein that is already persulfidated in one cysteine (−Cys-SSH) may function as a sulfur donor, transferring the terminal sulfur atom to a recipient thiol (−Cys-SH or glutathione), resulting in a new persulfidated molecule and the regeneration of the original cysteine thiol on the donor protein (Filipovic *et al*., 2018). The described 3-MSTs in *Arabidopsis thaliana*, known as sulfurtransferase 1 (STR1) and sulfurtransferase 2 (STR2), are recognized as important sulfurtransferases that contribute to the cellular pool of H₂S and sulfane sulfur (Fig. 1) (Selles *et al*., 2019; Moseler *et al*., 2021). This mechanism allows for the propagation and amplification of persulfidation signals across the proteome, potentially targeting specific protein networks. The involvement of persulfidated glutathione (GSSH) or other low-molecular-weight (LMW) persulfides (Vignane and Filipovic, 2023) as an intermediate sulfur donor further expands the complexity of this pathway, linking H_2_S signaling to the cellular glutathione pool.

In addition to direct enzymatic pathways to form persulfides, metalloprotein binding sites, particularly zinc fingers regions, emerge as prime targets for regulation by this modification (Fig. 1). A recent proteomic meta-analysis has gone even further and revealed that persulfidation of zinc finger proteins is a broad PTM impacting all classes of these cysteine-rich proteins across different species, suggesting a fundamental role of zinc finger proteins in protein persulfidation (Li *et al*., 2024). Zinc finger proteins are characterized by the coordination of one or more zinc ions (Zn^2+^) by cysteine and/or histidine (His) residues. The proximity of these thiol-carrying ligands to the metal ion makes them suitable to be targets for persulfidation. The persulfidation of the cysteine ligands in a zinc finger domain can directly affect the binding of the zinc ion, provoking rearrangements of the finger motifs. These conformational changes can also interfere with the protein’s ability to bind its partners, like DNA, RNA, or other proteins. Therefore, considering the heterogeneous function of the zinc finger in transcriptional regulation, DNA repair, and protein scaffolding, their modulation by persulfidation may have extensive biological consequences. Besides, how H_2_S directly persulfidates zinc finger proteins has been elucidated, with the process potentially facilitated by Zn²⁺ ions which can influence the reactivity of cysteine residues within these metalloproteins (Lange *et al*., 2019). This study revealed the critical role of Zn²⁺ in facilitating the direct persulfidation of zinc finger proteins by H₂S, indicating a crosstalk between metal ions and H₂S signaling (Fig. 1).

Other metals beside zinc are present in proteins and may also be modified by persulfidation. For example, H_2_S can interact with iron (Fe) in heme proteins. While Fe itself is not directly modulated by persulfidation, H_2_S could interact with heme iron or other cysteine residues around it, indirectly affecting the protein's function, such as in the case of cytochromes (Pietri *et al*., 2011). Similarly, other metal ions coordinated by cysteine residues, such as copper (Cu) or iron-sulfur clusters, could potentially be influenced by persulfidation of the coordinating ligands, leading to alterations in their redox properties or activities (Doman *et al*., 2023). However, a more complex possibility for the interaction of metal centers with H₂S or related sulfur species involves the generation of sulfur radical (HS^•^), which might then act as a reactive sulfur species capable of inducing persulfidation in other proteins (Fig. 1) (Kolluru *et al*., 2020).

The published data about protein persulfidation predominantly highlights direct H₂S reactions, enzymatic sulfur transfer by sulfurtransferases, and transsulfuration. Evidence specifically linking protein-metal center-mediated HS^•^ generation to downstream persulfidation of other proteins is less prevalent but remains a potentially important, albeit perhaps less common, mechanism. Further research employing advanced spectroscopic and trapping techniques would be needed to fully elucidate the contribution of this pathway to the overall landscape of protein persulfidation.

## The molecular mechanism of thioredoxin-mediated depersulfidation

**Fig. 2.**
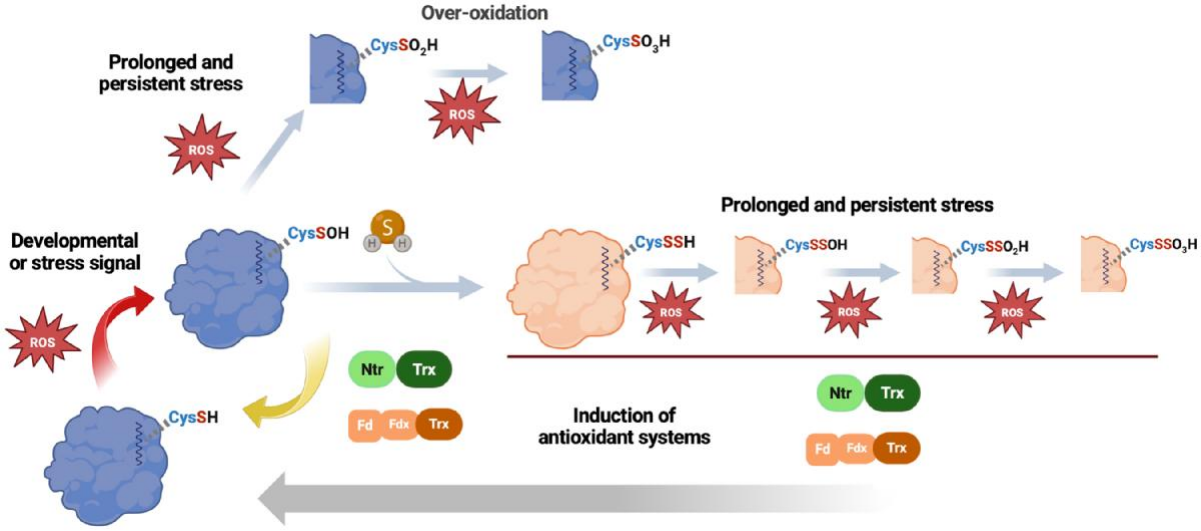
Mechanism of action for hydrogen sulfide (H_2_S) in protein protection against over-oxidation. The interplay between protein sulfenic acid (Cys-SOH) and protein persulfide (Cys-SSH) modifications is crucial for redox signalling. H_2_S reacts with sulfenylated Cys-SOH to form persulfidated Cys-SSH, which is a prerequisite for persulfidation. Sulfenylated residues can be reduced back to thiols by antioxidant systems, but under prolonged reactive oxygen species (ROS) stress can drive their further oxidation to irreversible sulfinic (Cys-SO_2_H) or sulfonic (Cys-SO_3_H) acid modifications. Under persistent oxidative stress conditions, persulfidated proteins can also be overoxidized to their corresponding perthiols (CysSSO_n_H) but these forms can be reverted to their reduced form by the action of antioxidant enzyme systems. Fd: ferredoxin, Fdx: flavodoxin, Ntr: NADPH thioredoxin reductase, Trx: thioredoxin. Created in BioRender. Aroca, A. (2025) https://BioRender.com/adrn71o.

Due to the multifaceted roles of protein persulfidation, its dynamic regulation necessitates efficient mechanisms for its turnaround. While the generation of protein persulfides has been extensively studied (Vignane and Filipovic, 2023), the mechanisms governing their removal, or depersulfidation have been less explored. Nevertheless, persulfidation and depersulfidation are equally vital for maintaining cellular redox homeostasis and the spatiotemporal dynamics of H_2_S signaling. In fact, the interplay between protein sulfenic acid (Cys-SOH) and protein persulfide (Cys-SSH) modifications represent a crucial aspect of redox signaling. For example, a comparative study performed in Arabidopsis revealed that a total of 1701 proteins were targets for both sulfenylation and persulfidation, which demonstrated an 82% identity between sulfenylome and persulfidome (Aroca *et al*., 2021b). However, this data may be underestimated because the Arabidopsis samples were very different in terms of developmental stage, tissue, or growth conditions, and new proteomic studies utilizing improved technologies have come out since this study. As highlighted, oxidative stress-induced sulfenylation can be a prerequisite for persulfidation. However, while sulfenylated residues can be reduced back to thiols by various enzymes, prolonged stress provokes persistent ROS, driving further oxidation to sulfinic (Cys-SO_2_H) or sulfonic (Cys-SO_3_H) acid, which are generally considered irreversible modifications. Conversely, under conditions of sustained oxidative stress, persulfidated residues can undergo further oxidation to perthiolsulfonic (Cys-SSOH), perthiolsulfinic (Cys-SSO_2_H), and perthiolsulfonic (Cys-SSO_3_H) acids. These oxidized forms can be efficiently reduced back to thiol groups by biological reductants, such as the thioredoxin (TRX) systems, in a manner similar to the reduction of persulfide groups (Fig. 2) (Gotor *et al*., 2019; Zaffagnini *et al*., 2019).

Recent investigations have highlighted the TRX system as a key player in the depersulfidation of proteins, providing a molecular basis for the transient nature of this modification (Vignane and Filipovic, 2023). In animal systems, the pivotal role of the TRX system in depersulfidation was demonstrated utilizing an improved tag-switch method (Wedmann *et al*., 2016), providing evidence that TRX acts as a depersulfidase, contributing to the intracellular protein persulfidation levels.

The specificity of thioredoxins is related with their distinct substrate recognition, which is not only determined by primary or tertiary structure, but is also strongly influenced by electrostatic similarity (Gellert *et al*., 2019). Thioredoxin-like proteins often possess a Cys-X-X-Cys active site that facilitates their redox activity. The enzymatic activity of thioredoxins shows a clear preference for persulfides, with recombinant thioredoxin demonstrating an almost 10-fold higher reactivity towards cysteine persulfide compared to cystine (Wedmann *et al*., 2016). Although the characterization of the biochemical parameters for the depersulfidation reaction have not been revealed for various thioredoxin-like proteins, in *Staphylococcus aureus*, three different thioredoxins exhibit varying catalytic efficiencies for reducing protein persulfides. This work demonstrated that TrxA (kcat=0.13 s⁻¹), TrxP (kcat=0.088 s⁻¹), and TrxQ (kcat=0.015 s⁻¹) can reduce protein persulfides on the model target pyruvate kinase (PykA) (Peng *et al*., 2018). Furthermore, different thioredoxins demonstrate distinct specificities by likely targeting largely non-overlapping sets of substrates in the cell.

However, the understanding of protein depersulfidation mechanisms in plants has lagged. Recently, the first comprehensive evidence for TRX-mediated depersulfidation in the plant kingdom was provided (De Brasi-Velasco *et al*., 2025). This work demonstrated that the mitochondrial thioredoxin o1 (TRXo1) in plants possesses depersulfidase activity, capable of removing sulfur from persulfidated proteins. Authors elegantly demonstrated that recombinant TRXo1 had the ability to reduce persulfidated cysteine residues in model substrates. The *in vitro* experiments revealed that TRXo1, in conjunction with the NADPH-dependent thioredoxin reductase, effectively catalyzed the removal of the extra sulfur atom from persulfidated proteins. This direct biochemical evidence established TRXo1 as an authentic depersulfidase in plants (Fig. 2). Further to the *in vitro* experiments, authors also demonstrated the physiological relevance of TRXo1's depersulfidase activity *in vivo* by a proteomic analysis of *trxo1* loss-of-function mutant plants (De Brasi-Velasco *et al*., 2025). Comparative proteomic analysis of these mutants and wild-type plants revealed a significant increase in the abundance of persulfidated proteins in the *trxo1* mutants. This accumulation of protein persulfides in the absence of TRXo1 strongly suggested that, under physiological conditions, TRXo1 plays a crucial role in the removal of these modifications within plant cells, particularly within the mitochondria where TRXo1 is localized. They demonstrated that the H_2_S-induced activation of certain mitochondrial enzymes activity from Arabidopsis, such as stromal ascorbate peroxidase (sAPX), dehydroascorbate reductase 1 (DHAR1), and monodehydroascorbate reductase 6 (MDAR6), is likely through persulfidation, and that this activation could be reversed by the action of the complete thioredoxin system, including TRXo1. This observation highlights the regulatory role of TRXo1 in modulating enzyme activity by controlling the persulfidation status of key proteins within the mitochondria. This research establishes a novel role for thioredoxins in plants, beyond their well-known disulfide reductase activity, and highlights a potentially conserved mechanism for regulating protein persulfidation across kingdoms of life. This suggests that the ability of thioredoxins to act as depersulfidase might be a fundamental and evolutionarily conserved mechanism for modulating H_2_S signaling and redox states in both animal and plant cells. Further research will undoubtedly focus on identifying other depersulfidases and fully characterizing the specific roles of different TRX isoforms in this crucial regulatory process across both kingdoms.

## Protein persulfidation function in metabolic and other fundamental biological processes

The analysis of 7 proteomic studies in the model plant *Arabidopsis thaliana* (Aroca *et al*., 2017a; Laureano-Marin *et al*., 2020; Jurado-Flores *et al*., 2021; Jurado-Flores *et al*., 2023a; Jurado-Flores *et al*., 2023b; Garcia-Calderon *et al*., 2023; De Brasi-Velasco *et al*., 2025) and those carried out in rice (*Oryza sativa subsp. japonica*) (Zhang *et al*., 2024), pepper (*Capsicum annuum*) (Muñoz-Vargas *et al*., 2024), and bean (*Phaseolus vulgaris*) (Matamoros *et al*., 2024) show that the persulfidation of proteins in plant systems is a ubiquitous post-translational modification, and the number of polypeptides currently identified as susceptible to persulfidation reaches 11 700 (see Dataset S1 at Zenodo, https://zenodo.org/records/16029996). In Arabidopsis, the most studied system under various physiological conditions such as nutritional deficit, drought stress, light regime, photorespiration, and induced autophagy, the number of proteins identified comes up to 7328. These studies indicate that this post-translational modification is broadly distributed across the plant proteome and suggest that persulfidation may exert an impact comparable to that of phosphorylation or glycosylation. The functional analysis of these identified proteins by the Kyoto Encyclopedia of Genes and Genomes (KEGG) annotations indicates that at least 20% are involved in general metabolic pathways such as glycolysis, citrate cycle (TCA cycle), carbon fixation by the Calvin-Benson cycle, or biosynthesis of amino acids (Table 1). Biosynthesis of secondary metabolites represents the second most represented functional pathway, with up to 12% of the total proteins. The overrepresentation of these processes indicates that H_2_S and protein persulfidation basally regulate both primary and energy metabolism of the cell in a manner similar to other redox modifications such as thiol/disulfide-based regulation in chloroplasts that turn on the enzymes of the Calvin-Benson cycle in a light dependent manner (Huang *et al*., 2023; Yoshida and Hisabori, 2023).

**Table 1.** Functional annotation of persulfidated proteins based on KEGG classification in *Arabidopsis thaliana* (see Dataset S1 at Zenodo, https://zenodo.org/records/16029996)

Term	Pathway name	No of proteins	%	Benjamini
ath01100	Metabolic pathways	1490	20.9	7.78×10^−31^
ath01110	Biosynthesis of secondary metabolites	872	12.3	8.27×10^−28^
ath03010	Ribosome	281	3.9	2.38×10^−23^
ath01200	Carbon metabolism	248	3.5	5.38×10^−42^
ath01230	Biosynthesis of amino acids	225	3.2	9.79×10^−42^
ath01240	Biosynthesis of cofactors	181	2.5	6.36×10^−13^
ath00010	Glycolysis/Gluconeogenesis	107	1.5	1.84×10^−16^
ath00270	Cysteine and methionine metabolism	94	1.3	2.52×10^−6^
ath01210	2-Oxocarboxylic acid metabolism	90	1.3	3.46×10^−12^
ath00620	Pyruvate metabolism	88	1.2	5.05×10^−14^
ath00520	Amino sugar and nucleotide sugar metabolism	87	1.2	2.90×10^−4^
ath03015	mRNA surveillance pathway	84	1.2	4.33×10^−4^
ath00480	Glutathione metabolism	83	1.2	3.51×10^−8^
ath03013	Nucleocytoplasmic transport	83	1.2	3.84×10^−7^
ath01250	Biosynthesis of nucleotide sugars	79	1.1	8.57×10^−7^
ath03018	RNA degradation	76	1.1	8.94×10^−3^
ath00230	Purine metabolism	75	1.1	1.83×10^−6^
ath00630	Glyoxylate and dicarboxylate metabolism	74	1.0	3.32×10^−14^
ath00710	Carbon fixation by Calvin cycle	69	1.0	1.09×10^−14^
ath00260	Glycine, serine and threonine metabolism	64	0.9	2.03×10^−10^
ath00020	Citrate cycle (TCA cycle)	59	0.8	5.59×10^−10^
ath01232	Nucleotide metabolism	58	0.8	9.74×10^−3^
ath03050	Proteasome	56	0.8	2.68×10^−9^
ath00030	Pentose phosphate pathway	55	0.8	2.03×10^−10^
ath00400	Phenylalanine, tyrosine and tryptophan biosynthesis	53	0.7	5.59×10^−10^

%: Percentage of persulfidated proteins respect to the total number of proteins of the pathway.

H_2_S enhances photosynthesis by increasing chlorophyll content, promoting chloroplast biogenesis, and up-regulating Rubisco activity. Both the Rubisco large subunit (RBCL) and the small subunits (RBCS) 1A, 1B, 2B, or 3B have been identified as susceptible to be persulfidated (Chen *et al*., 2011; Aroca *et al*., 2017a). The level of persulfidation of these proteins is also dependent on light regime, so it has been quantified that in darkness, these RBCS subunits 1A, 1B and 3B and enzymes of TCA and glycolysis, such as pyruvate dehydrogenase, succinate dehydrogenase (SDH1-1, SDH2-1), and several ATPase subunits are more persulfidated (Jurado-Flores *et al*., 2023b).

**Fig. 3.**
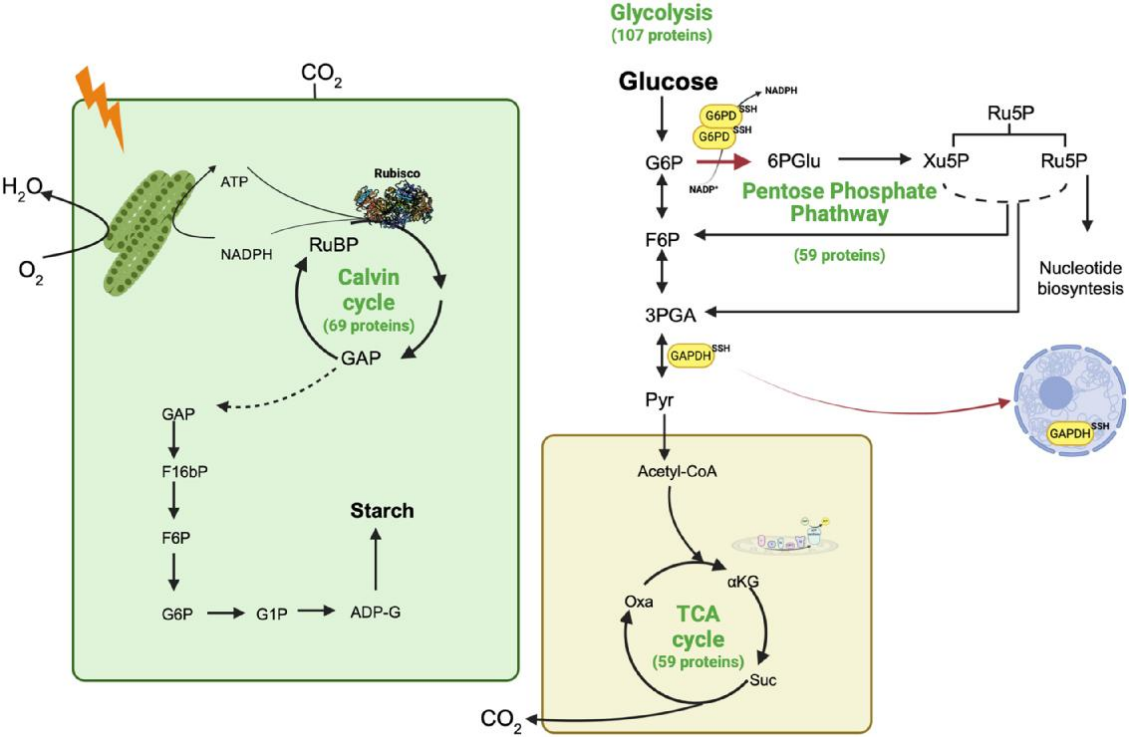
Overrepresented metabolic processes of persulfidated proteins based on KEGG annotation. Diagram shows the main metabolic pathways related to primary metabolisms and an example of two proteins whose modification affects the metabolic flow, glucose-6-phosphate dehydrogenase (G6PD) and glyceraldehyde-3-phosphate dehydrogenase (GAPDH), described in the text, is shown. The numbers between parenthesis indicate the identified persulfidated proteins, including isoforms, involved in each process, as indicated in Table 1. Chloroplast and mitochondria compartments are represented as boxes. NADPH: nicotinamide adenine dinucleotide phosphate (reduced form), RuBP: ribulose-1,5-bisphosphate, GAP: glyceraldehyde-3-phosphate, F16bP: fructose-1,6-bisphosphate, F6P: fructose-6-phosphate, G6P: glucose-6-phosphate, G1P: glucose-1-phosphate, ADP-G: ADP-glucose, 3PGA: 3-phosphoglycerate, Pyr: pyruvate, Oxa: oxaloacetate, αKG: alpha-ketoglutarate, Suc: succinate, TCA: tricarboxylic acid cycle, Ru5P: ribulose-5-phosphate, Xu5P: xylulose-5-phosphate, 6PGlu: 6-phosphogluconate. Created in BioRender. Romero (2025)  https://BioRender.com/5u3cp95.

Unlike conventional redox regulation based on reduction/oxidation of thiol residues, persulfidation also modulates the activity of enzymes and affects other features of the proteins, such as their location, polymeric state, or interaction with other proteins. This is the case of glyceraldehyde-3-phosphate dehydrogenase (GAPDH), in which treatment with exogenous H_2_S increases its enzymatic activity, and the persulfidation of the cytosolic isoform C1 of the GADPH (GAPC1)^C160^ residue regulates its subcellular localization from the cytosol to the nucleus, where it interacts with transcription factors to promote the expression of stress genes (Aroca *et al*., 2015; Aroca *et al*., 2017b; Kim *et al*., 2020). Another example of protein regulation of primary metabolism by H_2_S-mediated persulfidation is the glucose-6-phosphate dehydrogenases (G6PDs). G6PDs play a crucial role as the rate-limiting enzyme in the pentose phosphate pathway, producing NADPH for reductive biosynthesis and regulating cellular redox homeostasis. The persulfidation of the Cys^159^ of the cytosolic AtG6PD6 enhances its activity by altering the tetrameric structure of the protein, promote the affinity to NADP and protect the cysteine residue from oxidation in prolonged stress (Wang *et al*., 2023) (Fig. 3). The level of persulfidation of the cytosolic G6PD6 has been described as regulated by nutrient deprivation, being more persulfidated under nitrogen deprivation (Jurado-Flores *et al*., 2021).

H_2_S and persulfidation can also exert negative regulation on metabolic processes, significantly inhibiting the activities of key enzymes such as NADP-isocitrate dehydrogenase, NADP-dependent malic enzyme, and glutamine synthetase (Aroca *et al*., 2015; Munoz-Vargas *et al*., 2018; Munoz-Vargas *et al*., 2020). Therefore, protein persulfidation can serve to modulate and reprogram the primary and energy metabolism of the cell, depending on the physiological or environmental context of the plant.

**Fig. 4.**
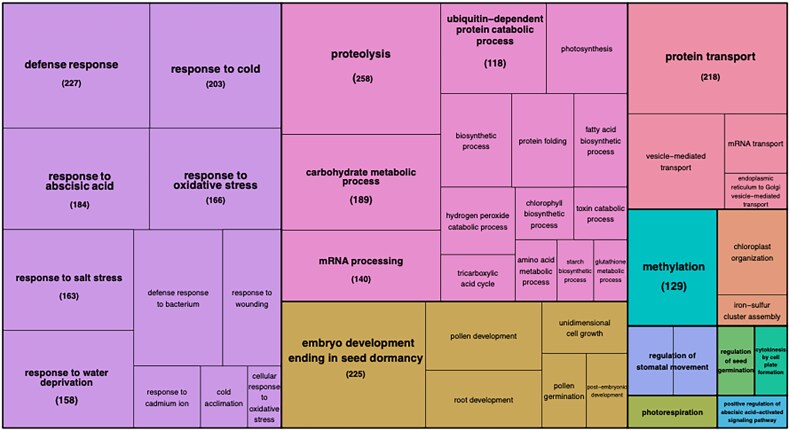
Functional classification of the Arabidopsis persulfidated proteins based on GO annotation by biological process. Tree-map representation with enriched biological process to which the proteins belong is shown. The colour arrangement illustrates the relationship within a hierarchy, and the number of persulfidated proteins in the highest hierarchy processes is indicated in parentheses. Box size represents the number of proteins.

In addition to its function in the regulation of primary and secondary metabolism of plants, functional analysis based on the annotation in gene ontology (GO) by biological processes highlights other relevant processes in plants, which are also regulated by persulfidation (Fig. 4). The main subgroups of persulfidated proteins belong to processes related to proteolysis, defense response, embryo development, protein transport, and response to cold, accounting for over 200 different proteins. Other processes regulated by H_2_S, which have been widely studied, also stand out, such as response to abscisic acid (ABA), response to oxidative stress, and response to water deprivation.

## Function in regulating proteolysis

Protein degradation is a vital cellular process that acts as a quality control and regulatory system, ensuring the proper functioning and health of cells. This process is crucial for maintaining cellular homeostasis and preventing the accumulation of damaged or misfolded proteins that could be toxic (McShane and Selbach 2022). Protein degradation serves several critical functions in plant cells, such as hormone control and signaling, light responses, senescence and nutrient remobilization, stress tolerance, and in developmental processes.

The group of persulfidated proteins classified in functional category ‘proteolysis’ contains members of the two main pathways responsible for protein degradation in eukaryotes: the autophagy pathway and the ubiquitin-proteasome system (UPS).

Autophagy in plant cells is precisely regulated by the developmental stage and changes in the environment. It is well established in plants that, under stress conditions, there is an induction of the autophagy process as a critical survival strategy (Signorelli *et al*., 2019). The mechanism of H_2_S signaling to regulate autophagy in eukaryotic cells was first described in Arabidopsis, observing that, under basal growth conditions, sulfide acts as a repressor of the process through protein persulfidation (Alvarez *et al*., 2012; Laureano-Marin *et al*., 2016, 2020; Gotor *et al*., 2022). The number of identified persulfidated proteins involved in the regulation of autophagy or in the process itself is very high and include the serine/threonine kinase TOR, its effectors proteins RAPTOR 1 and LST8, and several autophagy-related (ATG) proteins, including ATG2, 3, 5, 7, 11, 13, ATG18 and the cysteine protease ATG4 (Jurado-Flores *et al*., 2021; Jurado-Flores *et al*., 2023b). Induced-autophagy upon nutritional limitation either in dark-induced carbon starvation or nitrogen deprivation altered the level of persulfidation of the proteins ATG3 and ATG5 (Jurado-Flores *et al*., 2021; Jurado-Flores *et al*., 2023b). The underlying mechanism of the regulation of autophagy by H_2_S has been studied in more detail in the induction of autophagy by ABA, which increases concentration under abiotic stress conditions. Under basal growth conditions, the ATG4 protein is persulfidated at the catalytic residue Cys^170^, inhibiting its proteolytic activity which is essential for cleaving the C-terminal extension of ATG8, a key protein for the formation of autophagosomes. An increase in the intracellular level of ABA leads to a transient decrease in ATG4 persulfidation, which in turn favors the processing of ATG8, thereby allowing its lipidation and promoting autophagy progression (Laureano-Marin *et al*., 2020).

A similar mechanism has been proposed for the regulation of autophagy during endoplasmic reticulum (ER)-stress by H_2_S. In this stress condition, the core autophagy component ATG18 is reversibly persulfidated at Cys^130^, regulating its phospholipid-binding activity, essential for the elongation of phagophore membranes and the formation of the autophagosome (Aroca *et al*., 2021a). Persulfidation of this protein at Cys^78^ in the rice blast fungus *Magnaporthe oryzae* also regulates autophagy and is crucial for fungal virulence (Hu *et al*., 2025).

Many members of the ubiquitin-proteasome system (UPS) are also extensively modified by persulfidation and impacts all groups of enzymes including the Ub-activating enzyme E1, Ub-conjugating E2, and Ub-ligases E3 of the HETC, U-box, single RING-finger, and multi subunits RING-finger/Cullin types. The role of H_2_S and persulfidation in the regulation of proteins of UPS have already been described; upon nitrogen deficiency, the E3 ligase more axillary branches 2/oresara 9 (MAX2/ORE9) reduces its persulﬁdation level by 30-fold. MAX2 belongs to the F-box leucine-rich protein family and is a central component in the strigolactone and karrikin signaling cascade (Nelson *et al*., 2011). In addition, the level of persulfidation of the RING/U-box RING domain ligase 1 (RGLG1) and 5 (RGLG5) were increased. These E3 ligases negatively regulate the drought stress response as they promote the degradation of ABA-related phosphatases (PP2Cs) or the MAPKKK18 which mediates the MAPK cascade and plays important roles in ABA signaling and drought tolerance (Wu *et al*., 2016; Jurado-Flores *et al*., 2021; Yu *et al*., 2021). In *Solanum lycopersicum* (tomato), the transcription factor WRKY71 interacts with the E3 ligase *Botrytis* susceptible 1 interactor-related gene 3 BRG3 that regulates WRKY71 ubiquitination and degradation. When BRG3, a RING-type domain-containing protein, is persulfidated at Cys^206^ and Cys^212^ its ubiquitination activity is reduced and thereby stabilizes WRKY71, that acts as a repressor of fruit ripening (Sun *et al*., 2023).

The RING domain E3 ligase comprises a domain signature where His and Cys residues bind zinc atoms. These internal Cys residues can be targeted for persulfidation, destabilizing the domain and consequently its activity (Lange *et al*., 2019). Given that many of the target proteins of E3 ligases are involved in abiotic stress, it is not surprising that H_2_S signaling and protein persulfidation play a very important regulatory role in biological processes such as drought, salinity, and heat stress. In fact, pretreatment with H_2_S induces resistance to drought stress in several plant species, in which persulfidation of the UPS and proteosome enzymes increases (Jurado-Flores *et al*., 2023a; Zhang *et al*., 2024; Carrillo *et al*., 2025).

At least, 41 subunits of the 20S and 26S proteasome are also susceptible to be modified by persulfidation. In fact, under light, the most overrepresented GO process is the proteasomal protein catabolic process which have been shown to be induced under oxidative stress by enhancing the switch from 26S to 20S (Jurado-Flores *et al*., 2023b).

All the components of the constitutive photomorphogenesis 9 (COP9) signalosome complex, CSN1-8, are also able to be persulfidated. There are not many studies on the effect of H_2_S on the regulation of photomorphogenesis, but it has been observed that H_2_S regulates the elongation of the hypocotyl in *Setaria* P. Beauv. Red light can also induce the expression of cysteine desulfhydrases and the production of H_2_S (Liu *et al*., 2019). The constitutive photomorphogenesis 1 (COP1) protein has not been detected as persulfidated, but has been detected in proteins that directly interact with it, such as COP1-interactive proteins CIP1, CIP4, and CIP7. The role of H_2_S and persulfidation in signalosome regulation remains unknown and opens the door to future studies.

## Involvement in defense responses

Numerous persulfidated proteins have relevant functions in various plant defense processes and in response to abiotic stresses, oxidative stress, and hormonal changes (Fig. 4). The generic biological process subgroup ‘defense response’ comprises 230 proteins related with plant immunity, including 10 nucleotide-binding site (NBS) and leucine-rich repeat (LRR) protein receptors involved in the detection of diverse pathogens, as well as 1-aminocyclopropane-1-carboxylate oxidases (ACO2 to 5); cysteine proteinase inhibitors (CYS1 to 5); many endochitinases and endo-1,3-beta-glucosidases; and well characterized proteins involved in immunity such as enhanced disease susceptibility 1 (EDS1), jasmonate ZIM domain-containing protein 13 (JAZ13), BRI1-associated receptor kinase 1 (BRK1), suppressor of npr1-1 constitutive 1 (SNC1) or coronatine-insensitive protein 1 (COI1). The effect of persulfidation on the function of these proteins has not been studied but it is known that H_2_S induces their accumulation. Proteomic analysis of spinach seedlings treated with H_2_S shows an increase in the abundance of defense related proteins such as ethylene-forming enzyme, NBS-LRR domain resistance proteins, and resistance to *Pseudomonas syringae* pv. maculicola (RPM)-interacting proteins, highlighting the role of H_2_S in the immune response (Chen *et al*., 2014). Infection of Arabidopsis leaves with virulent *Pseudomonas syringae* (*Pst*) strain DC3000 increased H_2_S production by inducing the expression and activity of cysteine desulfhydrases, LCD and DCD. LCD and DCD overexpression lines were more resistant to biotic stress, inducing the expression of defense proteins such as EDS1 and various pathogen-related (PR) proteins (Shi *et al*., 2015). Reduction in H_2_S synthesis in Arabidopsis leaf mitochondria impaired the induction of the stomatal closure triggered by the bacterial PAMP flagellin (flg22), and plants were more susceptible to bacterial surface inoculation (Pantaleno *et al*., 2025).

Proteins and enzymes involved in the response to oxidative stress and redox homeostasis are overrepresented in proteomic studies in *Arabidopsis thaliana* and crop species performed above (see Dataset S1), and includes ascorbate peroxidases (APXs), catalases (CATs), glutathione S-transferases (GSTs), methionine sulfoxide reductases (MSRs), peroxidases (PERs), superoxide dismutases (SODs) and TRXs. The effects of persulfidation on the activity of these enzymes have been well-documented. A common characteristic among most of these enzymes is their nature as metalloproteins. They all contain a co-factor, one of heme, Fe-S clusters, or a metal cation like Cu, Fe, or Zn which, as described above, play a crucial role in promoting the formation of persulfides. This is the case for APXs, CATs, MSRs, PER, and SODs, which are widely represented in all persulfidomes carried out to date. Persulfidation of the Arabidopsis AtAPX1^C32^ and tomato SlAPX1^C168^ induces their enzymatic activity but has no effect on the APX enzyme of *Phaseolus vulgaris* (common bean) (Aroca *et al*., 2015; Li *et al*., 2020; Matamoros *et al*., 2024). The increased activity of AtAPX by H_2_S/persulfidation is reversible by the action of TRXs, which reverse the formation of the persulfide residue to thiol (De Brasi-Velasco *et al*., 2025). Under non-photorespiratory conditions in Arabidopsis, H_2_S treatment induces protein persulfidation and protects from oxidative stress, inducing CAT activity and reducing the H_2_O_2_ content in leaves and reactive oxygen species in guard cells (Garcia-Calderon *et al*., 2023). *In vivo* H_2_S pretreatment does not induce changes in APX activity or H_2_O_2_ content, but it does prevent H_2_O_2_ accumulation in a subsequent drought situation (Jurado-Flores *et al*., 2023a; Zhang *et al*., 2024; Carrillo *et al*., 2025). However, an opposite effect was observed in tomato, where persulfidation of the SlCAT^C234^ decreased its enzymatic activity (Li *et al*., 2020).

Persulfidation of the SOD enzyme has been detected in Arabidopsis and common bean, and, although the impact of persulfidation on SOD enzymatic activity remains unclear, previous research indicates that it inhibits the tyrosine nitration of SOD, which safeguards SOD from irreversible nitro-oxidative modifications (Matamoros *et al*., 2024).

In general, it is widely known that H_2_S relieves oxidative stress in numerous stress conditions such as salt stress, metal toxicity, and drought, or prolongs postharvest storage by inducing the activity of various antioxidant enzymes (Hu *et al*., 2014; Mostofa *et al*., 2015; Jia *et al*., 2016; Ahmad *et al*., 2020). Although this effect has been observed at both the transcriptional and enzymatic levels, the persulfidation of these key defense enzymes may be essential for the catalytic function of the enzyme, as well as for its protection against over-oxidation. 2-Cys peroxidases PrxA and PrxB, which operate in peroxide signaling and detoxification, are recurrently identified in persulfidomes. These proteins react with H_2_O_2_ to form a sulfenic acid intermediate that can either form a disulfide bond, which commits the enzyme to its peroxidase cycle, or it can react with peroxide again to produce a sulfinic acid that inhibits both PrxA and B (Kriznik *et al*., 2020). Over-oxidation of PrxA and PrxB is associated with a functional switch from peroxidase to chaperone activity (Puerto-Galan *et al*., 2013). Since the persulfidation of cysteine residue is associated to an oxidative event, it is plausible that persulfidation may be a critical mechanism in regulating the activity of these proteins.

## Role of persulfidation on abscisic acid and cold responses

There is currently a large body of evidence on the crosstalk of H_2_S with ABA in plant stomatal movement and has been extensively reviewed (Xuan *et al*., 2020; Aroca *et al*., 2021b; Cui *et al*., 2025; Jaiswal *et al*., 2025; Mohammadbagherlou *et al*., 2025). At least 5 of the ABA receptors, pyrabactin resistance 1(PYR1) and pyrabactin resistance-like (PYL1, 2, 8 and PYL9), have been identified as susceptible to persulfidation. In addition, main components of the initial core for the perception and transduction of ABA, like several protein phosphatases 2C, such as hypersensitive to ABA (HAB)1 (PP2C16), HAB2 (PP2C7), ABA-hypersensitive germination 3 (PP2C37), protein phosphatase 1 (PPH1) (PP2C57), ABA-insensitive 2 (ABI2) (PP2C77), and sucrose non-fermenting 1-related protein kinases (SnRK) that work downstream in ABA signaling (SnRK2.2, SnRK2.4, SnRK2.6/OST1, or SnRK1.1), were also identified in several persulfidomes (Aroca *et al*., 2017a; Jurado-Flores *et al*., 2021; Jurado-Flores *et al*., 2023b). It is well established that H_2_S increases ABA-induced stomatal closure in several species, suggesting that it is a component of the ABA signaling pathway in guard cells (Scuffi *et al*., 2014; Pantaleno *et al*., 2021). In fact, the effect of ABA-induced persulfidation on specific cysteine residues of several components of this signaling pathway has been demonstrated, such as the H_2_S-producing L-cysteine desulfhydrase 1 DES1^C44/C205^ enzyme that induces persulfidation of respiratory burst oxidase homolog protein D RBOHD^C825/C890^ and the protein kinase SnRK2.6/OST1^C131/C137^ (Chen *et al*., 2020; Shen *et al*., 2020). Likewise, the persulfidation of the transcription factor ABI4^C250^ has been demonstrated to promote *MAPKKK18* transactivation, triggering downstream signaling and affecting seed germination and growth (Zhou *et al*., 2021).

Proteins related to cold stress responses are also one of the enriched persulfidated protein groups (Fig. 4). H_2_S has been shown to alleviate cold stress at different growth stages of the plant, including in seedling, during senescence and fruit ripening, and postharvest storage, as recently reviewed (Cui *et al*., 2025). H_2_S fumigation or the loss-of-function of the *DES1* gene have been shown to regulate the expression of genes involved in cold and chilling stress response, such the *COLD-REGULATED* (*COR15a* and *COR15b)* genes, through up-regulation of the *MITOGEN-ACTIVATED PROTEIN KINASE* 4 (*MPK4*) gene expression (Alvarez *et al*., 2012; Du *et al*., 2017; Wu *et al*., 2024). In addition to regulating their expression, these proteins are also susceptible to be persulfidated (Du *et al*., 2021; Garcia-Calderon *et al*., 2023; Jurado-Flores *et al*., 2023b; De Brasi-Velasco *et al*., 2025). Glycine-rich RNA-binding protein (GRP) has been characterized to function as RNA chaperones in cold adaptation (Kwak *et al.*, 2011), and at least nine GRPs have also been identified in several persulfidome analyses.

## Developmental processes regulated by H_2_S

Although there are not many studies, there is some evidence that H_2_S regulates various processes of plant development such as embryogenesis, seed dormancy and germination (Baudouin *et al*., 2016; Lorenz *et al*., 2018).

A significant number of persulfidated proteins are classified within the biological function of ‘embryo development ending in seed dormancy’, ‘root development’, and ‘pollen development and germination’ (Fig. 4). In Arabidopsis, a collection of 510 embryo-defective genes (*EMB*) are required for embryo development and have a phenotype in development due to loss-of-function (Meinke, 2020). These genes perform diverse functions associated with proteins involved in RNA binding and modiﬁcation, or they are pentatricopeptides-repeat (PPR) proteins and plastid-localized ribosomal proteins, which are overrepresented in the 510 *EMB* genes (Meinke, 2020). At least 32 of the persulfidated proteins in this group are designated as EMB proteins, such as EMB514, DOMINO 1, EMB140, or the GTP-binding protein EMB2001 (Lahmy *et al.*, 2004). This category also includes proteins that regulate the cell cycle, such as cyclin-dependent kinase A-1 (CDKA1/CDC2A), several PPR proteins, such as GRP23 and EMB1796, or with diverse functions, including the histone-binding protein multicopy suppressor of IRA1 (MSI1), cell wall extension 3, and the acetyl-CoA carboxylase 1 (ACC1). So far, the effect of persulfidation on enzyme activities and interactions with other proteins is unknown, but it seems clear that H_2_S must have a regulatory role in this process.

The most direct evidence for the involvement of H₂S in plant embryogenesis comes from studies on the *ETHYLMALONIC ENCEPHALOPATHY 1 (ETHE1)* and the *SULFURTRANSFERASE 1 (STR1)* genes in Arabidopsis. ETHE1 is a sulfur dioxygenase enzyme responsible for detoxifying H₂S in mitochondria, and plants with a non-functional *ETHE1* gene have embryos that arrest at the early heart stage of development and are ultimately not viable (Holdorf *et al*., 2012). A small amount of ETHE1 sulfur dioxygenase activity is enough for embryo survival, but development is severely delayed (Krussel *et al*., 2014). The protein STR1 is a mitochondrial 3-mercaptopyruvate sulfurtransferase enzyme involved in H₂S generation, coupled to thioredoxin, or its detoxification, coupled to ETHE1 (Selles *et al*., 2019). STR1 mutation also results in shrunken seed phenotype, related with delayed embryo development (Mao *et al*., 2011; Moseler *et al*., 2021). Recently, the persulfidation level of both proteins has been shown to be regulated by the action of mitochondrial thioredoxin TRXo1, suggesting that mitochondrial H_2_S homeostasis may play a role in regulation of the embryogenesis (De Brasi-Velasco *et al*., 2025).

In addition to embryo development, H_2_S also affects and regulates root development, since exogenous application increases the number and length of lateral roots (Zhang *et al*., 2009; Fang *et al*., 2014; Wang *et al*., 2024). The exogenous application of H_2_S in tomato induces the expression of cell cycle regulatory genes such as the CDKA1, which is also persulfidated, promoting root development through nitric oxide and indole-3-acetic acid dependent signaling (Zhang *et al*., 2009; Fang *et al*., 2014). H_2_S generated by cysteine desulfhydrase induces auxin accumulation and alleviates the root growth inhibition induced by osmotic stress or phosphate starvation (Xiang *et al*., 2023; Liu *et al*., 2024). Studies on the interaction between sulfur and hormone regulation in root development have also recently been demonstrated. Persulfidation of cytokinin oxidase/dehydrogenases (CKX2) at Cys^62^ enhances its activity and reduces cytokinin levels, affecting the root system architecture (RSA) (Wang *et al*., 2024). Recently, the persulfidation of the SnRK1^C419/C430/C505^ energy sensor has been shown to increase its activity and promote lateral root formation in peach (*Prunus persica*) (Wu *et al*., 2025). Both studies highlight how persulfidation of key proteins ultimately influences root development.

## Contribution of H_2_S on methylation functional process

Despite the lack of evidence regarding the H_2_S-regulated protein and DNA methylation levels in plants, it is interesting that there are numerous persulfidated proteins classified in the functional methylation process. Among these proteins are the cobalamin-independent methionine synthase 1 (MS1) and the isoforms MS2 and MS3, several S-adenosyl-L-methionine-dependent methyltransferases, and histone-lysine N-methyltransferases such as absent small and homeotic disks protein 1 (ASH1), ATP-dependent helicase (ATXR), suppressor of variegation 3-9 homolog protein 3 (SUVH3) and the COMPASS-like component transducin/WD40 repeat-like superfamily protein (WDR5A). This observation suggests that H_2_S could regulate the activities of these enzymes and alter methylation in DNA or histones. Recent studies have shown that pretreatment of plants with low concentrations of H_2_S protects the plant against subsequent stress processes, suggesting that H_2_S may act as a chemical inducer of priming (Jurado-Flores *et al*., 2023a; Zhang *et al*., 2024; Carrillo *et al*., 2025). Epigenetic modification of DNA or proteins may be one of the priming mechanisms that may carry short- or long-term defense and acclimation responses to biotic and abiotic stress in plants (Harris *et al*., 2023). The effect of H_2_S on epigenetic mark modification in plants and other eukaryotic systems remains unproven, despite some indirect suggestions in eukaryotes (Spezzini *et al*., 2023).

## Conclusion

H_2_S signaling in plants is a prominent research area with exponential research growth since H_2_S is crucial in the regulation of essential plant processes from metabolism and development programs, to environmental responses and immunity. The studies performed to date have shown that the primary mechanism of H_2_S signaling, protein persulfidation, is ubiquitous and has been shown to be a dynamic and reversible modification that alters and regulates protein conformation and function, as well as enzyme activities and protein-protein interaction. There is no doubt that future studies will uncover new processes in planta that are regulated or induced by H_2_S and will open the door to obtaining more resilient crops in the face of the challenges of climate change and food demand.

## Data Availability

Dataset S1 is available at Zenodo, https://zenodo.org/records/16029996 (Romero *et al*., 2025).
